# Dispersal Pathways and Genetic Differentiation among Worldwide Populations of the Invasive Weed *Centaurea solstitialis* L. (Asteraceae)

**DOI:** 10.1371/journal.pone.0114786

**Published:** 2014-12-31

**Authors:** Renée L. Eriksen, José L. Hierro, Özkan Eren, Krikor Andonian, Katalin Török, Pablo I. Becerra, Daniel Montesinos, Liana Khetsuriani, Alecu Diaconu, Rick Kesseli

**Affiliations:** 1 Department of Biology, University of Massachusetts Boston, Boston, Massachusetts, United States of America; 2 Instituto de Ciencias de la Tierra y Ambientales de La Pampa (Consejo Nacional de Investigaciones Científicas y Técnicas-Universidad Nacional de La Pampa), Santa Rosa, La Pampa, Argentina; 3 Biyoloji Bölümü, Fen-Edebiyat Fakültesi, Adnan Menderes Üniversitesi, Aydın, Turkey; 4 Environmental Studies Department, De Anza College, Cupertino, California, United States of America; 5 Centre for Ecological Research (MTA ÖK), Vácrátót, Hungary; 6 Departamento de Ecosistemas y Medio Ambiente, Facultad de Agronomía e Ingeniería Forestal, Pontificia Universidad Católica de Chile, Santiago, Chile; 7 Centre for Functional Ecology, Departamento de Ciências da Vida, Faculdade de Ciência e Tecnologia da Universidade de Coimbra, Coimbra, Portugal; 8 Institute of Botany, Ilia State University, Tbilisi, Georgia; 9 Institute of Biological Research, Biological Control Laboratory, Iasi, Romania; Natural History Museum of Denmark, Denmark

## Abstract

The natural history of introduced species is often unclear due to a lack of historical records. Even when historical information is readily available, important factors of the invasions such as genetic bottlenecks, hybridization, historical relationships among populations and adaptive changes are left unknown. In this study, we developed a set of nuclear, simple sequence repeat markers and used these to characterize the genetic diversity and population structure among native (Eurasian) and non-native (North and South American) populations of *Centaurea solstitialis* L., (yellow starthistle). We used these data to test hypotheses about the invasion pathways of the species that were based on historical and geographical records, and we make inferences about historical relationships among populations and demographic processes following invasion. We confirm that the center of diversity and the native range of the species is likely the eastern Mediterranean region in the vicinity of Turkey. From this region, the species likely proceeded to colonize other parts of Europe and Asia via a slow, stepwise range expansion. Spanish populations were the primary source of seed to invade South America via human-mediated events, as was evident from historical records, but populations from the eastern Mediterranean region were also important. North American populations were largely derived from South America, but had secondary contributors. We suggest that the introduction history of non-native populations from disparate parts of the native range have allowed not just one, but multiple opportunities first in South America then again in North America for the creation of novel genotypes via intraspecific hybridization. We propose that multiple intraspecific hybridization events may have created especially potent conditions for the selection of a noxious invader, and may explain differences in genetic patterns among North and South America populations, inferred differences in demographic processes, as well as morphological differences previously reported from common garden experiments.

## Introduction

Inferring processes and historical relationships from contemporary patterns is one of the primary goals of population genetics, and that information can help shed light on important environmental issues such as invasive species management. In particular, the invasion history and the evolutionary processes that contribute to the success of a species may be postulated by understanding the genetic diversity and similarities among populations from the native and non-native ranges. One evolutionary process that population genetics routinely illuminates is intraspecific hybridization, which along with other factors such as altered selective regimes in new habitats, may have significant impacts on the competitive capabilities and performance of invasive species [1]. The importance of hybridization is not new to the study of evolutionary biology, though it has not always been applied to invasive species biology. Anderson and Stebbins [2], [3], [4] and Mayr [5] noted the importance of hybridization to the evolutionary history of species more than 50 years ago and, indeed, speciation as a result of hybridization events and adaptation to new environments have been repeatedly documented, particularly in the plant family, Asteraceae [6], [7], [8], [9].

The emergence of superior competitors after repeated opportunities for intraspecific hybridization resulting from the globalization of plant species would therefore seem to be an expected outcome. Through globalization, individuals from vastly different parts of the native range are repeatedly moved to new habitats, eliminating former geographic barriers to reproduction, and enabling individuals from disparate populations to interbreed. Increasing attention to the role of hybridization and admixture in colonizing species has highlighted the importance of heterosis and the increased evolutionary potential of admixed populations in initiating non-native invasions [10], [11], [12], [13]. In this study, we compare patterns of contemporary genetic diversity to look for evidence of intraspecific hybridization, to infer population structure, and to test hypotheses of invasion pathways of *Centaurea solstitialis* L. (yellow starthistle). This rangeland weed of the Asteraceae is relatively innocuous [14] and even rare in its native range [15] but has become a noxious invasive species in much of its non-native range [14], [16], [17], [18].

The history of *C. solstitialis* in non-native regions is remarkably well understood. Maddox [19] cites Hendry's work in the 1930s analyzing seeds present in the brick of Spanish and Mexican buildings from 1700s and 1800s. Hendry found no *C. solstitialis* seeds in mission buildings constructed prior to 1824, but did find seeds from those constructed after that date. Robbins et al. 1951 (cited in [19]) noted that *C. solstitialis* was a common contaminate of alfalfa seed. The earliest report of alfalfa cultivation in California was in Marysville, 1851 [20], and the first herbarium record of *C. solstitialis* was collected in 1869 from Oakland, California [17]. According to Gerlach [20], there are no records of alfalfa introduced to California from any other country aside from Chile until 1898. The species, however, is not native to Chile, and is believed to have been introduced to South America from Spain via the same vector [20]. It was probably introduced to central Argentina circa 1870 [21]. The geographic center of what is considered the species' native range is Turkey [14] and several subspecies of *C. solstitialis* have been described throughout the native range, four of which are found in Europe [22] and three of which are found in Turkey [14]. Uygur et al. [14] theorized that the *C. solstitialis* found in North America consists of a mixture of these subspecies, though to our knowledge no studies distinguish different subspecies in the non-native regions.

Though the ecology and management of *C. solstitialis* has been the subject of a wide range of articles, there have been only four published genetic studies of this important invader. Sun and Ritland [23] used allozyme markers to conclude that the species is a pollinator-dependent obligate outcrosser in North America. Sun [24] found high levels of genetic diversity and a general lack of inter-population divergence in allozymes (GST  = 0.095) among populations from the western United States. Sun concluded it was likely that there were multiple introductions of *C. solstitialis* to the United States from a common seed source. Eriksen et al. [25] assessed phenotypic and neutral genetic variation among native and non-native accessions grown in a common garden experiment. They found greater phenotypic variation partitioned among regions than neutral genetic variation, suggesting local adaptation. More recently, Dlugosch et al. [26] used single nucleotide polymorphism (SNP) data contained in cDNA sequences obtained from next-generation sequencing to trace the invasion routes of *C. solstitialis*. They were largely able to confirm Gerlach's [20] work on invasion pathways, but also found significantly more heterozygosity in non-native populations than in native populations. When they looked for population substructure among populations from the native and non-native range, they found no structure distinguishing their native and non-native samples, suggesting there have been multiple introductions from the native range to multiple parts of the invaded range [26].

Here, we seek to extend these genetic studies at greater sampling depth by looking at seven microsatellite (also known as simple sequence repeats or SSR) loci designed from an EST database. We sampled 520 individuals from multiple populations from the non-native region, including California, U.S.A., Argentina, and Chile, as well as multiple populations from the putative native region, including those in Spain, Turkey, the Republic of Georgia, Armenia, Romania, Hungary, and Uzbekistan, encompassing the most ambitious collection of populations of this global invader to date. The scope of our collections and the hypervariability of the SSRs, allows us to compare population genetic patterns at multiple levels. Because the goal of this work was to conduct a global survey of the species, samples from local populations were small. The assayed loci from our specimens allow us to compare levels of diversity within and among regions of the world to assess the relative frequency and source of dispersal events in certain regions. That is, comparing the proportion of alleles that are unique to certain regions (private alleles) and calculating genetic distances (F_ST_, Nei's genetic distance, and Shannon's diversity index) allows us to hypothesize whether populations from geographically distant regions share a common history, while characterizing the portion of overall genetic diversity that is partitioned within and among different regions (AMOVA) allows us to detect evidence of population substructure. Together, these data elucidate possible critical differences in invasion dynamics within North and South America and provide a sketch of the global invasion history of the species.

## Materials and Methods

### Collections, Planting and DNA Extractions

**Figure 1:**
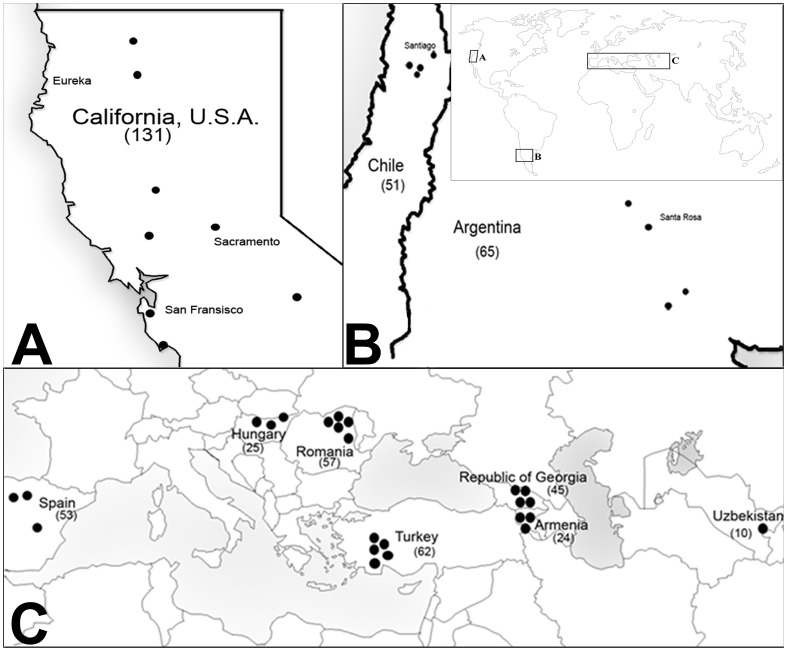
Approximate population collection localities. Map A: California (U.S.A., North America); Map B: Chile and Argentina (South America); Map C: Spain, Hungary, Romania, Turkey, Georgia, Armenia, and Uzbekistan (Eurasia). The number of individuals from each country is given in parentheses.

Seed samples were collected from 39 wild populations in Europe, South America and the U.S. (Fig. 1) as described in Hierro et al. [21]. Heads were collected from up to 30 different individuals randomly chosen from across each site. A single seed (technically an achene) from at least 10 different individuals was planted in small 2 cm2 pots and grown in the greenhouse at the University of Massachusetts Boston, U.S.A. Leaf tissue from rosettes of individuals that germinated and survived was harvested after about 4 weeks. We extracted DNA from the 520 individuals using one of three methods: the Qiagen DNA extraction kit (Qiagen, Valencia, California, USA), the FastDNA extraction kit (MP Biomedicals, Solon, Ohio, U.S.), or standard CTAB methods.

### EST-SSR Primer Design and DNA Fingerprinting

We screened the EST database of the Composite Genome Project (http://compgenomics.ucdavis.edu/) and identified all ESTs with 2 bp and 3 bp SSRs with ten or more repeats and all 4 bp SSRs with five or more repeats using the program MiSA [27]. We used PRIMER3 [28] to design primers for the 34 ESTs containing the longest tetra- and tri-nucleotide repeats. We then attached a fluorescent 17-bp M13 tag onto the 5′ end of the forward primer. PCR conditions were optimized, and seven primer pairs were chosen for their ease of amplification and scoring; these seven loci contain trinucleotide repeats (Table 1; [25]).

**Table 1:** EST-SSR loci.

SSR Locus	Repeat Motif	Primer Sequence		PCR	Size Range (bp)	No. Alleles	Average H_o_ Per Population	Average H_e_ Per Population	CGP Accession No.
9840	(ATC)11	F:	CAGGAAACAGCTATGACATAAAGCCGTGGTTTCGTTG	HS 50	136–211	13	0.49	0.55	CNSM9840.b1_O12.ab1
		R:	CAAGTGTCGTTCGCTTTCAC						
10599	(ACA)11	F:	CAGGAAACAGCTATGACAGATGGCGACGGATAACATC	TD 58	235–259	9	0.42	0.60	CNSM10599.b1_M10.ab1
		R:	GCTGCAGGCAAGGTCTTAAA						
11320	(AGA)13	F:	CAGGAAACAGCTATGACGAAACCAGCAGAGGAGCAAG	50	190–244	16	0.64	0.66	CNSL11320.b1_P21.ab1
		R:	CAGCAGAATTTCCGGTTCAT						
15790	(AGA)12	F:	CAGGAAACAGCTATGACCACGGGATGACGAAAGACTC	HS 50	176–239	18	0.46	0.66	CNSM15790.b1_K11.ab1
		R:	TTTGTGTTAACGCCAGAGGA						
22883	(ACA)11	F:	CAGGAAACAGCTATGACCGGCAGATAGACCATCCTTC	HS 50	146–215	24	0.76	0.74	CNSS22883.b1_E10.ab1
		R:	TCCTTTGCATCCATTCTTCC						
27745	(ATC)13	F:	CAGGAAACAGCTATGACCAAAACCCAGCATCAAGACC	50	322–379	16	0.66	0.69	CNSS27745.b1_B01.ab1
		R:	GCAGAGGAGTTTGTGCATGA						
1459	(GAA)12	F:	CAGGAAACAGCTATGACCGAACCTCCTTTCAGCATTC	TD 60	157–203	16	0.45	0.68	CNSM1459.b1_F06.ab1
		R:	CCCAGTAGCCTCAAGACCAA						

The EST-SSR loci names, repeat motifs, as well as the primer sequences with the M13 tag attached to the 5′ end of the forward primer are given. Also given are the optimized PCR protocol (HS  =  hot start; TD  =  touchdown) and annealing temperatures, the size range of the amplicons, the number of alleles found in this study, and the Composite Genome Project (CGP) accession number. The observed (H_o_) and expected (H_e_) heterozygosity per population is also given for each primer. No observed heterozygosity differed significantly from the expected (χ^2^>0.996 for all loci)

Polymerase chain reactions were performed in 25 µl volumes with 1–10 ng/µl gDNA, 0.1 µM of the forward primer, 0.4 µM of the reverse primer, and 0.3 µM of a fluorescently labeled M13 primer, 1x reaction buffer, 2.5 mM combined dNTPs, 3 mM MgCl_2_, and 1.25 units of Taq polymerase (Promega GOTaq Flexi DNA Polymerase). We used different amplification protocols, all featuring variations of the following protocol: 5 minutes denaturation at 94°C, 35 cycles of 94°C for 30 seconds, optimized annealing temperature for 30 seconds (Table 1), 71°C for 30 seconds, followed by a final extension at 71°C for 5 minutes. Some primers required a touchdown protocol with the following cycles: 3 minutes at 95°C; 10 cycles of 30 seconds at 94°C, 30 seconds at 60–58°C and 45 seconds at 72°C, optimized annealing temperature (Table 1) decreasing by 1°C per cycle, followed by 30 cycles of 30 seconds at 94°C, 30 seconds at 50°C, 45 seconds at 72°C, and followed by a final 20 minutes at 72°C.

The PCR products were assayed on a 3100-Avant Genetic Analyzer (ABI). We used Peak Scanner software for analysis (ABI). Peaks were assigned numbers by Peak Scanner based on the 400HD ROX size ladder which approximated the length of the amplicon and each allele call was confirmed individually. A subset of individuals was re-sampled at some loci for confirmation.

### Estimates of Genetic Diversity and Genetic Divergence

To control for sample size variation, we scored private alleles and calculated allele richness (As) using a rarefaction method in HP-Rare [29]. We used GenAlEx 6.5 [30], [31] to calculate observed and expected heterozygosity and Wright's F-statistics, as well as Nei's genetic distances and pairwise Shannon diversity indices. Due to the number of pairwise comparisons involving 39 populations, we visualized the patterns using principle components analysis (PCA) calculated via covariance matrix on standardized data in GenAlEx 6.5. Welch tests, a non-parametric equivalent to the ANOVA, were performed with SPSS v. 21, Inc (IBM).

We examined linkage disequilibrium via χ^2^ tests and a likelihood ratio test for unknown gametic phase in Arlequin v. 3.1.1 [32], [33], [34], and population substructure in Arlequin v. 3.1.1 and STRUCTURE 2.3 [35]. STRUCTURE 2.3 was run through the front-end version of the program at University of Massachusetts, Boston, as well as through the CBSU Web Computing Interface at Cornell University for K = 2–42 for 39 populations in the study plus 3 as suggested by the manual using a MCMC burn in of 10,000 steps, and 10,000 iterations. A priori population information was input into the program, and this information was incorporated by the simulation. The correlated allele frequency model was used, as well as the admixture model of ancestry in which the degree of admixture, alpha, was inferred from the data. All other parameters were at the default settings. The most likely K value was evaluated using the methods described by Evanno et al. [36] in MS Excel 2007 and confirmed using STRUCTURE Harvester [37]. The simulations were subsequently rerun with the same parameters and 100,000 iterations for K = 2–8 to ensure the parameters reached equilibrium and the most likely K value was again determined using the same methods. Two replicate simulations from K = 7 and 8 were removed from the data set because the runs did not converge. Graphical displays of STRUCTURE results were exported from the program and modified in Adobe Photoshop CS3 for clarity.

## Results

From 40,406 sequences representing 22,917 different contigs derived from *C. solstitialis* and characterized as part of the Composite Genome Project, we identified all 2 bp and 3 bp SSRs with ten or more repeats (101 and 64 ESTs respectively) and all 4 bp SSRs with five or more repeats (187 ESTs); these data are available at http://www.genetics.umb.edu/. We designed primers for 34 of the longest SSRs with sufficient 5′ and 3′ flanking space. Eighteen were polymorphic within a sample subset, but because the SSRs were isolated from an EST database, it was common to find unexpected introns in the final amplicon that made scoring difficult. We confirmed the presence of introns by sequencing [38] and excluded most primer pairs that amplified these gene regions from further study. Seven pairs of trinucleotide SSRs markers remained and the number of alleles for each locus ranged from 9–24 alleles (Table 1). Linkage disequilibrium among loci within populations was assessed and χ^2^ values were not significant for nearly all pairs of loci in all populations. Linkage disequilibrium was detected for some pairs of loci in some populations using a likelihood ratio test in Arlequin v. 3.1.1, but this was not consistent among populations and was likely caused by historical founding events in invaded regions or consanguineous matings and unknown local population structure (data available at http://www.genetics.umb.edu/). Average observed heterozygosity (H_o_) per population for each marker ranged from 0.42–0.76, and did not differ significantly from expected heterozygosity (H_e_) in 34 of the 39 populations (Table 1).

### Allele Frequency Analyses

We used these EST-SSR markers to assay 4–24 individuals from 39 populations within 10 countries. The average number of alleles per locus found in each population ranged from 1.86–7.86, but when normalized for population size using rarefaction, the allelic richness ranged from A_s_ = 1.75–4.30 (Table 2). Populations from Turkey had higher values ranging from A_s_ = 3.93–4.30. Populations from the non-native range (California, Argentina, and Chile), also had high numbers of alleles, ranging from A_s_ = 3.40–3.68, 3.30–3.92, and 3.82–4.10 alleles per locus respectively. Private alleles, or alleles that are unique to particular populations, were found in 12 populations from California, Argentina, Chile, Spain, Turkey, Armenia, Romania, and Uzbekistan (Table 2). These populations had 1–2 private alleles with frequencies of 0.03–0.13; the highest concentration of private alleles was found in populations from Turkey and Armenia. Despite relatively high sampling, we found only three private alleles in all North and South American populations and only one of those was found more than once in a single population (Chi2 from Chile; Table 2).

**Table 2:** Population collections from each country and basic statistics.

Country	Pop.	No. Samples	Average No. Alleles Per Locus	A^s^	No. Private Alleles	Freq Private Allele	H_o_	H_e_	F
**CA, U.S.A.**	**CAL1**	19	6.57	3.59			0.57	0.70†	0.20
	**CAL2**	13	5.29	3.58	1	0.04	0.55	0.69	0.21
	**CAL3**	17	5.57	3.68			0.63	0.70	0.13
	**CAL4**	15	6.29	3.52			0.66	0.67	0.01
	**CAL5**	11	5.43	3.48			0.59	0.65	0.09
	**CAL6**	24	5.29	3.47			0.50	0.64	0.18
	**CAL7**	23	6.29	3.64			0.68	0.70	0.03
	**CAL8**	7	3.86	3.40			0.49	0.62	0.22
**Argentina**	**ARG1**	19	6.86	3.92			0.68	0.74	0.05
	**ARG2**	14	5.29	3.30			0.60	0.64	0.08
	**ARG3**	17	6.00	3.58	1	0.04	0.45	0.70†	0.35
	**ARG4**	15	6.29	3.89			0.65	0.73	0.13
**Chile**	**CHI1**	5	4.43	3.85			0.64	0.67	0.05
	**CHI2**	14	5.71	3.82	1	0.12	0.53	0.71	0.25
	**CHI3**	15	6.57	4.10			0.57	0.75†	0.23
	**CHI4**	17	6.86	3.94			0.63	0.75	0.15
**Spain**	**SPA1**	11	2.86	2.49			0.35	0.53	0.29
	**SPA2**	23	6.71	3.88			0.68	0.75	0.10
	**SPA3**	19	5.57	3.48	1	0.03	0.61	0.66	0.06
**Turkey**	**TUR1**	20	6.86	3.93	1	0.06	0.67	0.73	0.09
	**TUR2**	15	7.86	4.30			0.73	0.78	0.08
	**TUR3**	11	6.71	4.12	1	0.05	0.59	0.74†	0.20
	**TUR4**	16	6.71	4.02	2	0.10, 0.13	0.67	0.74	0.09
**R. of Georgia**	**GEO1**	19	5.43	3.29			0.60	0.65	0.09
	**GEO2**	12	4.71	3.47			0.63	0.69	0.08
	**GEO3**	4	3.00	2.91			0.50	0.51	0.04
	**GEO4**	9	3.86	3.22			0.68	0.63	−0.10
**Armenia**	**ARM1**	11	5.00	3.38	1	0.05	0.58	0.63	0.08
	**ARM2**	6	4.29	3.40	2	0.08, 0.08	0.45	0.59	0.25
	**ARM3**	7	5.00	3.76	2	0.08, 0.08	0.43	0.67	0.37
**Romania**	**ROM1**	11	4.00	3.08			0.47	0.60	0.28
	**ROM2**	9	4.14	3.20			0.47	0.64†	0.26
	**ROM3**	16	5.86	3.82	1	0.03	0.52	0.74	0.31
	**ROM4**	8	4.00	3.15			0.60	0.61	0.03
	**ROM5**	13	4.29	3.23			0.40	0.63	0.33
**Hungary**	**HUN1**	7	1.86	1.75			0.26	0.31	0.16
	**HUN2**	10	3.29	2.70			0.43	0.57	0.25
	**HUN3**	8	3.29	2.84			0.42	0.59	0.34
**Uzbekistan**	**UZB1**	10	4.00	2.83	1	0.10	0.50	0.49	−0.02

† Significant deviation of observed from expected heterozygosity across all loci.

The population code, the number of individual samples from that population, the average number of alleles per locus and allelic richness (A^s^) are given. The number of private alleles, if any, as well as the frequency of those private alleles in that population is also provided. Values of observed (H_o_) and expected heterozygosity (H_e_) across all loci, significant deviations from the H_e_ (†) based on a P value (Bonferroni corrected), and Wright's fixation index (F) are also given for each population.

Gene diversity or expected heterozygosity (He) within populations was generally high with values averaging 0.65 and ranging from 0.31–0.78. There were no differences in average heterozygosity across all loci among native or non-native populations (Welch1, 35.2 = 0.56, P = 0.46) or among the populations from Eurasia, North America, and South America (Welch2, 17.4 = 0.54, P = 0.59).

Wright's Fixation Index [39], [40] was also calculated for each population across all loci. Most F values were close to zero as expected under random mating for an obligate outcrossing species (Table 2); however, a few populations ranged up to 0.37, suggesting the presence of local inbreeding or null alleles.

### Genetic Distance and Population Structure Analyses

**Figure 2:**
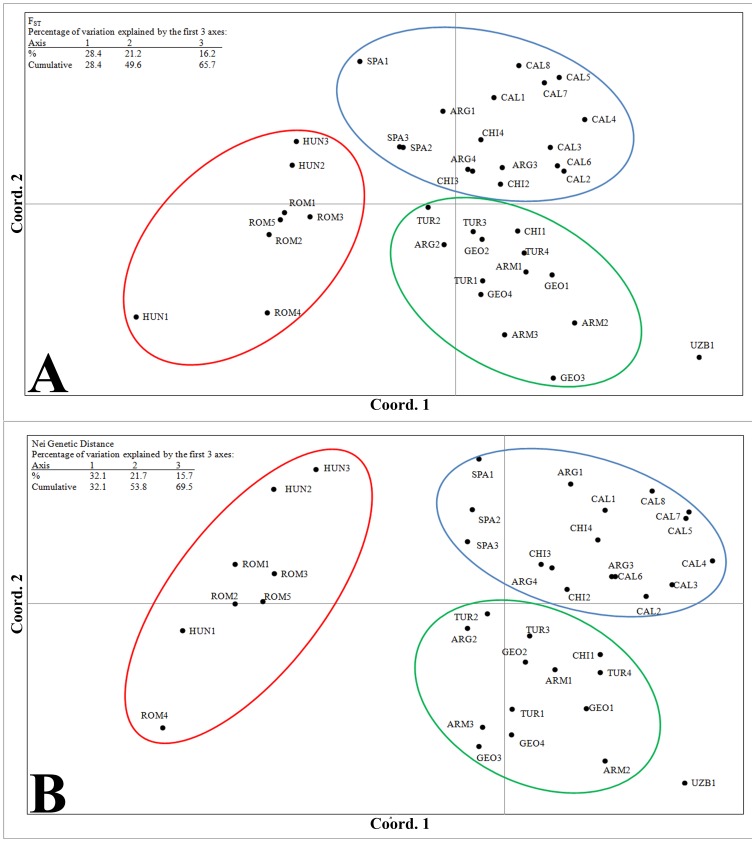
Principle components analysis plots. Each population is plotted based on FST (A) and Nei's Genetic Distance (B) for all populations. Three distinct groups were identified and circled; Romania-Hungary (red), Turkey-Armenia-Georgia (green) and California-Chile-Argentina-Spain (blue). Note: one population from Chile and one from Argentina cluster with the Turkey-Armenia-Georgia group.

We calculated pairwise F_ST_, Nei's genetic distance, and Shannon's diversity index for all pairs of populations (data available at http://www.genetics.umb.edu/) and for populations grouped by country (Table 3) to understand the genetic relationships among populations. Due to the number of pairwise comparisons involving 39 populations, we visualized the patterns using principle components analysis (PCA, Fig. 2). The three distance statistics revealed similar patterns of diversity among all the populations, though some minor differences in the rank order of these distances between pairs of populations were apparent. For example, all three statistics identified two populations in California as most similar (Cal5 and Cal7: F_ST_ = 0.013, Nei D = 0.055; Shannon SHUA  = 0.040). However, for Wright's F_ST_ values, a population from Hungary (Hun1) and one from Uzbekistan were most distinct (F_ST_  = 0.404), while both Nei's genetic distance and Shannon diversity indices identified a different pair, one population from Romania (Rom4) and another from Spain (Spa1) (Nei D = 2.182, SHUA  = 0.573) as most distinct (http://www.genetics.umb.edu/). Regardless of the distance statistic used, the PCA showed three groups of populations. Populations from Hungary and Romania formed one distinct group, populations from California, Argentina, Chile, and Spain formed a second, and populations from Turkey, the Republic of Georgia, and Armenia formed a third (Fig. 2). The population from Uzbekistan is an outlier of this latter group. One population from Argentina and one from Chile clustered with the Turkey, Republic of Georgia, and Armenia, eastern Mediterranean group. The California, Argentina, Chile, and Spain group of the PCA plots could be further subdivided as both the Spanish and California populations each formed distinct clusters. The first two axes of the PCA explained a similar amount of variation (49.6%–53.8%) regardless of the distance statistic used.

**Table 3:** Genetic distance.

	CA, USA	Argentina	ChiLE	SpaIn	TurKey	Georgia	Armenia	Romania	Hungary	Uzbekistan
**CA, USA**	-	0.268	0.244	0.548	0.372	0.430	0.500	0.864	0.745	0.516
**Argentina**	0.033	-	0.137	0.362	0.262	0.413	0.443	0.637	0.667	0.646
**Chile**	0.031	0.017	-	0.433	0.357	0.449	0.357	0.670	0.790	0.537
**Spain**	0.061	0.040	0.048	-	0.667	0.593	0.666	0.655	0.855	1.204
**Turkey**	0.042	0.027	0.038	0.063	-	0.280	0.357	0.605	0.740	0.356
**R. of Georgia**	0.056	0.051	0.056	0.071	0.038	-	0.270	0.635	0.977	0.378
**Armenia**	0.065	0.056	0.048	0.080	0.047	0.044	-	0.803	0.842	0.363
**Romania**	0.082	0.059	0.065	0.065	0.059	0.074	0.090	-	0.483	1.113
**Hungary**	0.090	0.077	0.089	0.095	0.082	0.112	0.107	0.065	-	1.701
**Uzbekistan**	0.122	0.131	0.121	0.182	0.099	0.114	0.101	0.178	0.228	-

Nei's Genetic Distance (D) is shown above the diagonal and Wright's F_ST_ values of genetic distance are given below the diagonal for each comparison among groups of populations within each country.

**Figure 3:**
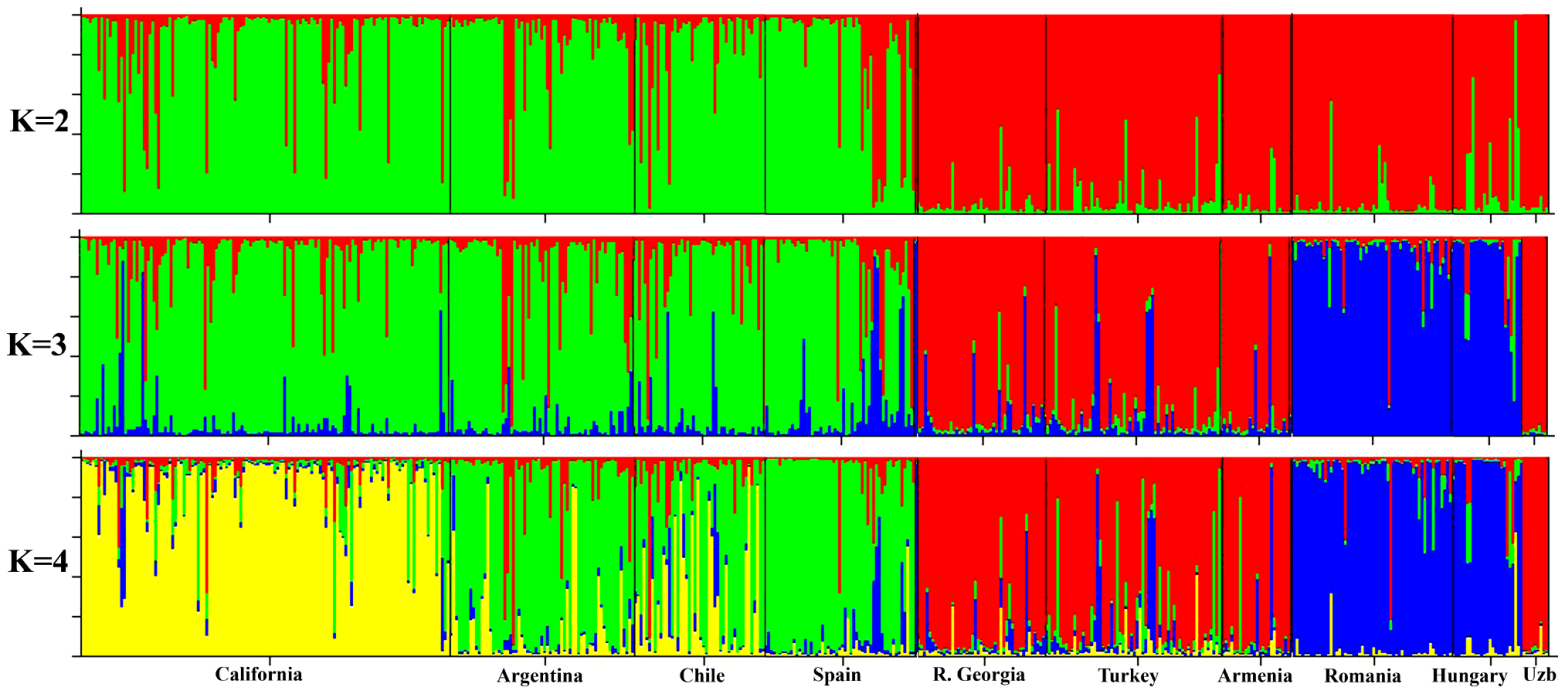
STRUCTURE analysis plot for K = 2, K = 3, and K = 4. According to the method described by Evanno et al., the true value of K is 4. Uzb  =  Uzbekistan.

STRUCTURE analyses displayed similar patterns in global population structure as the PCA. When K = 2, the populations are roughly subdivided into the Americas plus Spain and the Eurasian groups. Individuals from Spain are noteworthy as they are geographically European in origin, but fall mostly into the group with individuals from non-native populations in North and South America. When K = 3, Eurasian populations are subdivided into populations from Hungary and Romania, and populations from Turkey, Georgia, Armenia, and Uzbekistan. When K = 4, populations from California are distinguished from other non-native populations in South America and Spain (Fig. 3). Based on the methods described by Evanno et al. [36], the most likely value of K is 4 (Table 4).

**Table 4:** STRUCTURE true K Evanno et al. statistics.

K	Replicates	Mean L(K)	Stdev L(K)	Mean L'(K)	Mean |L''(K)|	ΔK
**2**	10	−13164	1.52			
**3**	10	−12779	10.80	385.04	19.38	1.80
*4	10	−12376	2.70	403.02	184.56	68.25
**5**	10	−12157	8.13	218.46	30.73	3.78
**6**	10	−11969	5.65	188.11	57.15	10.11
**7**	8	−11850	15.60	133.04	95.49	6.12
**8**	8	−11788	13.42	93.30		

The results of Evanno et al. calculations for K values of 2–8. The clear modal ΔK is for simulations in which K = 4 and is denoted by an asterisk (*).

### Differences Among Non-Native Regions

Differences among populations from the non-native regions (California, Argentina, and Chile) were also detected. AMOVA analysis [32], [33], [34] characterizing genetic structure within and among populations from these countries showed no significant differentiation among the populations of Argentina and Chile (Φ_CT_ <0, P = 0.68), but did detect significant differentiation among those in California and those in Argentina (Φ_CT_  = 0.04, P = 0.002) and in Chile (Φ_CT_  = 0.04, and P = 0.002). Thus, Californian populations appear distinct from South American populations even though alfalfa records and F_ST_ values suggest they have a shared history.

## Discussion

The present work provides a more comprehensive assessment of the genetic diversity, population structure, and relationships among native and non-native populations of the global invader *C. solstitialis* with a wider geographical scope than previous studies. Many global invaders, including *C. solstitialis*, exhibit remarkable differentiation between their native and non-native populations [14], [16], [21], [25], [41], [42]. Population genetic studies offer essential information for understanding the mechanisms operating behind the differentiation, such as intraspecific hybridization, as well as for inferring historical relationships such as source populations, dispersal routes, and demographic processes.

The Eurasian native *Centaurea solstitialis* is a relatively recent introduction to North and South America, but it has quickly established itself as one of the most aggressive invaders in large areas of its new range [43], [44]. Several studies have documented differences among the native and non-native populations, specifically in plant densities in the field [14] and in seed starch content and seedling growth for seeds originating from different regions [45]. Recently, common garden experiments found significant phenotypic differentiation in non-native populations for traits likely contributing to fitness and invasive capabilities. Eriksen et al. [25] compared individuals from two native (Turkey and Republic of Georgia) and two non-native (Argentina and California) regions and showed that non-native individuals had larger leaves, were taller (both regions), and flowered earlier (California only) than individuals from native regions. In addition, quantitative differentiation exceeded neutral genetic differentiation for many traits suggesting recent and rapid evolution. Graebner et al. [46] found that seedling mass was greater for plants from California than for those from Spain, particularly when in competition with other grassland species. Hierro et al. [47] showed that plants from Argentina achieved higher densities, greater plant size, and higher survivorship than those from Turkey and that these differences are affected by environmental conditions. Further, Montesinos et al. [48] recently demonstrated incipient reproductive isolation occurring between native and non-native populations based on reduced seed set when individuals from Californian seed stock were pollinated by individuals from Spain. These studies suggest an underlying genetic basis for the phenotypic differences found in native and non-native regions and the likely role of natural selection and rapid evolution in shaping these differences. The present study confirms this underlying genetic differentiation and uses contemporary patterns of population diversity and substructure to dissect the invasion pathways and events that contributed to the invasion success of this species.

As they disperse across the globe, there has often been speculation that non-native species will experience severe bottlenecks during their introduction and establishment [49]. Many populations of *C. solstitialis*, however, appear to possess extensive genetic diversity in their non-native range; this result was first reported by Sun [24] using isozyme data, and later confirmed and extended by Dlugosch et al. [26] using SNP data. Here, we were able to confirm these high levels of genetic diversity within non-native regions, but also were able to elucidate unique world-wide patterns in diversity. While Dlugosh et al. [26] found significantly higher H_o_ in non-native regions than native regions, we found no significant difference in H_o_ nor H_e_ between these regions nor among the continental groups of populations (North America, South America, Eurasia). We did, however, find great heterogeneity for gene diversity (H_e_), allelic richness (A_s_) and private allele values among populations in Eurasia. Populations from the eastern Mediterranean region and particularly the populations in Turkey had the highest levels of gene diversity (average of populations H_e_ = 0.75), highest rarified allelic richness (average of populations A_s_ = 4.1) and, along with Armenia, the highest number of private alleles (4 and 5 respectively). The populations of this eastern Mediterranean region (Turkey, Armenia and the Republic of Georgia) cluster together in our STRUCTURE analysis (Fig. 3) and form the central hub of the PCA based on F_ST_ and Nei's genetic distances (Fig. 2). These results are consistent with the documented high morphological diversity, prevalence of taxonomically distinct subspecies and the hypothesis that the eastern Mediterranean region is the likely origin and center of diversity for the species [14].

In contrast to these high gene diversity, allelic diversity and private allele statistics in the eastern Mediterranean region, we found low gene diversity, low allelic diversity and few private alleles in populations from Spain, Hungary, and Uzbekistan. Interestingly, low gene diversity was previously noted within Spanish populations [19], [26] and has conservation implications in Hungary where it is a protected species [15]. These populations in the geographically peripheral regions sampled in this study cluster into three separate and distinct groups within the PCA surrounding the core group from eastern Mediterranean: Spanish populations cluster with populations from the Americas, Hungarian populations cluster with neighboring Romanian populations, and the single population from Uzbekistan is an outlier. These groups essentially form the spokes diverged from that core, eastern Mediterranean group. Low diversity in these populations is likely indicative of the pattern found in the peripheral populations of many species and is predicted in stepwise range expansion models involving recurrent bottlenecks [50], [51].

STRUCTURE analysis shows subdivisions within the global population, with non-native American populations generally clustering with Spanish populations, and also suggests evidence of admixture with the cluster of populations from Turkey, Georgia, and Armenia (Fig. 3). Populations from Turkey, Georgia, and Armenia are distinct from populations in Hungary and Romania in our STRUCTURE analysis as well as our PCA based on genetic distances, and this is consistent with Eurasian population structure found in previous studies [26]. It is likely that the divergence of Hungarian and Romanian populations from other Eurasian populations is due to isolation by distance. Together, these data paint a complex evolutionary history for this species with Eurasian populations likely expanding from the core Mediterranean region east to Asia (Uzbekistan), north to central Europe (Romania and Hungary) and west to Spain, with time for populations from each of these regions to evolve independently.

Evidence of the invasion pathways for *C. solstitialis* out of Eurasia to South America and later to North America has been previously presented. By examining literature assessing records of contaminated alfalfa seed lots, Gerlach [20] concluded that Spain was the source of Chilean populations of *C. solstitialis*. He also concluded that Chile in turn was likely the sole source of the Californian populations prior to the early 1900s, a time when the species was already considered a common weed and invader of fields and roadsides in California. The SNP data from Dlugosch et al. [26] as well as the low genetic distance values obtained in our study between the populations of California and those of Chile and Argentina support Gerlach's hypothesis about the primacy of Chilean sources for the California populations. Dlugosch et al. [26] further agreed that Spain was the probable source of seed for the South American populations. However, levels of differentiation, population structure, and gene diversity found in this study suggest that, while Spain may have been a primary source, it was not likely the sole source of seed for the non-native regions examined here. The situation is clearly more complicated and other native regions may also have been important sources of seed initially or via secondary introductions. Allelic richness and average gene diversity were lower in Spain (average of populations A_s_ = 3.3; H_e_ = 0.65) than in any of the American regions (average values for the three different regions were: A_s_ = 3.5–3.9; H_e_ = 0.67–0.72). Pairwise comparisons for genetic differentiation between American populations and multiple Eurasian countries were often low. Indeed, Nei's D and F_ST_ values averaged across populations were lower in the American and Turkish comparisons than for the American and Spanish ones (Table 3). Such low values may indicate multiple and perhaps repeated introductions to South America from Eurasia. Few, if any, of these introductions appear to have come from the peripheral regions of Uzbekistan, Hungary, and Romania which were genetically distinct from populations in the Americas (Table 3). STRUCTURE analysis when K = 2 showed a clear division between American plus Spanish versus Eurasian populations, again highlighting Spain as a major source of the non-native founding populations (Fig. 3). The PCA shows Spanish populations associated with, though distinct from the American populations (Fig. 2). However, the South American populations as a group appear as close to the major eastern Mediterranean cluster as to Spain. Indeed, one population from Argentina and one from Chile are embedded within that Eastern Mediterranean group (Fig. 2). These data suggest that populations from outside of Spain were also important contributors to the initial invasion of the Americas.

Further dissection within STRUCTURE (K = 4) and close inspection of the PCA help to reveal the final stage of global expansion examined in this study. Californian populations are clearly distinct from those in South America, and while the AMOVA shows no significant difference between populations in Chile and those of Argentina (Φ_CT_ <0, P = 0.68), the populations of California are significantly differentiated from both (Φ_CT_  = 0.04, P = 0.002 for both comparisons). There are also differences in population structure within these non-native regions. The F_ST_ values among populations in California are all low, but tend to be higher among populations in both Chile and Argentina. Low substructure in California was also noted by Sun [24]. The allelic richness is also more uniform in California. These statistics suggests the modes of range expansion may have been different in the two non-native regions. In North America, historical records [17] and genetic data suggest that *C. solstitialis* probably expanded its range rapidly from diverse founding populations. In contrast, the South American region and particularly populations from Argentina show more substructure and more differentiation among populations, possibly suggesting a less rapid range expansion. This less rapid range expansion could be due to dispersal barriers such as climatic differences and altered selective pressures in Argentina. Eriksen et al. [25] showed that several morphological and developmental traits distinguished Argentinian populations from Californian and Turkish populations in common garden experiments. The wet summers of Argentina verses the dry summer climates found throughout much of the Mediterranean and California may have provided a crucial selective change and altered the dispersal dynamics in this region.

Together, these data paint a complex evolutionary history for this species with Eurasian populations expanding from the core Mediterranean region east to Asia (represented in this study by Uzbekistan, though the eastern limit of the species' range is not well documented), north to central Europe (Romania and Hungary) and west to Spain. Each of these regions evolved independently and retained the signatures (low gene diversity, low allele richness) of bottlenecks within introduced populations. The initial expansion of this species out of the core Mediterranean region may have involved human-mediated events, however we suggest that more natural dispersal could be responsible for the observed patterns in Eurasia. The clearly human-mediated invasion to the Americans likely involved more rapid and multi-step processes with the initial influx of seed coming from Spain to South America and from there to California, U.S.A. These initial events were likely followed by additional introductions to South America and also to California from the eastern Mediterranean center of diversity increasing the genetic diversity in these non-native regions beyond that of the initial introductions from Spain. Certain individuals from California, Argentina, and Chile, have high posterior probabilities of being from Eurasia (Fig. 3), but populations have clearly differentiated from their native counterparts. Neutral evolutionary processes such as admixture events, range expansion, isolation by distance, minor local bottlenecks and subsequent genetic drift, as well as non-neutral processes such as local adaptation and selection have all likely altered the allele frequencies in the Americas enough to differentiate them from Eurasian populations. Admixture from multiple sources followed by recombination and selection may be the explanation for the larger plants, greater seed size and altered life history traits in non-native accessions from California and South America detected in common garden experiments [47].

## Conclusion

Multiple introductions of an invasive plant can create a “melting pot” of genetic diversity derived from a wide range of habitats and geographically isolated populations from the native region [11]. Intraspecific hybridization and recombination within this melting pot of genetic diversity can give rise to novel genotypes, creating populations with “high evolutionary potential” [11], [49]. Within this context, natural selection driven by the new abiotic and biotic pressures of the non-native habitat can probably act quickly to create aggressive and noxious invaders. This process is not wholly different from many classic breeding programs which create diverse populations through composite crosses followed by selection in repeated cycles [52]. These circumstances are also similar to conditions that Mayr [5] described as optimal for “genetic revolutions” and subsequently for rapid evolutionary change. While his emphasis was on founder populations and speciation, Mayr as well as Anderson and Stebbins [2], [3], [4] clearly noted that other factors such as hybridizations, rearranged gene pools, novel habitat can drive rapid evolutionary change. South American *C. solstitialis* populations have experienced a history of introductions from multiple sources of the native range, and this melting pot of rearranged genomes was, along with new introductions from native regions, a major source of populations that have now colonized North America. Thus, admixture events have occurred multiple times between diverse populations and subspecies in the non-native regions, and may have created especially potent conditions for the selection of a serious invader. Several studies have used common garden experiments to document morphological differences between native and non-native populations that may indicate evidence for adaptive morphological changes between native and non-native populations of *C. solstitialis* [25], [45], [46], [47]. All these differences among native and non-native populations seem to be the outcome of a set of propitious conditions for “genetic revolutions” and “high evolutionary potential”, which potentially played a critical role on the development of invasive ability in the American non-native range of this species.
